# 1-Hour OGTT Plasma Glucose as a Marker of Progressive Deterioration of Insulin Secretion and Action in Pregnant Women

**DOI:** 10.1155/2012/460509

**Published:** 2012-04-10

**Authors:** Alessandra Ghio, Giuseppe Seghieri, Cristina Lencioni, Roberto Anichini, Alessandra Bertolotto, Alessandra De Bellis, Veronica Resi, Emilia Lacaria, Stefano Del Prato, Graziano Di Cianni

**Affiliations:** ^1^Section of Metabolic Diseases and Diabetes, Department of Endocrinology and Metabolism, AOUP Pisa, University of Pisa, Ospedale Cisanello, Via Paradisa, 56124 Pisa, Italy; ^2^Department of Internal Medicine, Spedali Riuniti, 51100 Pistoia, Italy; ^3^Department of Diabetes and Metabolic Diseases, Livorno Hospital, 57100 Livorno, Italy

## Abstract

Considering old GDM diagnostic criteria, alterations in insulin secretion and action are present in women with GDM as well as in women with one abnormal value (OAV) during OGTT. Our aim is to assess if changes in insulin action and secretion during pregnancy are related to 1-hour plasma glucose concentration during OGTT. We evaluated 3 h/100 g OGTT in 4,053 pregnant women, dividing our population on the basis of 20 mg/dL increment of plasma glucose concentration at 1 h OGTT generating 5 groups (<120 mg/dL, *n* = 661; 120–139 mg/dL, *n* = 710; 140–159 mg/dL, *n* = 912; 160–179 mg/dL, *n* = 885; and ≥180 mg/dL, *n* = 996). We calculated incremental area under glucose (AUC_gluc_) and insulin curves (AUC_ins_), indexes of insulin secretion (HOMA-B), and insulin sensitivity (HOMA-R), AUC_ins_/AUC_gluc_. AUC_gluc_ and AUC_ins_ progressively increased according to 1-hour plasma glucose concentrations (both *P* < 0.0001 for trend). HOMA-B progressively declined (*P* < 0.001), and HOMA-R progressively increased across the five groups. AUC_ins_/AUC_gluc_ decreased in a linear manner across the 5 groups (*P* < 0.001). Analysing the groups with 1-hour value <180 mg/dL, defects in insulin secretion (HOMA-B: −29.7%) and sensitivity (HOMA-R: +15%) indexes were still apparent (all *P* < 0.001). Progressive increase in 1-hour OGTT is associated with deterioration of glucose tolerance and alterations in indexes of insulin action and secretion.

## 1. Introduction

Pregnancy is characterized by a complex endocrine-metabolic adaptation process including impaired insulin sensitivity, increased *β*-cell function, moderate elevation of blood glucose levels, particularly following the ingestion of a meal, and changes in the circulating levels of free fatty acids, triglycerides, cholesterol, and phospholipids [1]. These changes do not reflect a pathological condition; rather, they represent a necessary and indispensable adaptation to meet the energy demand of the foetus and to prepare the maternal organism for delivery and lactation. However, in 3–5% of pregnant women this physiologic adaptation [2] becomes abnormal and gestational diabetes may develop. Therefore, a condition that should result in healthy growth of the foetus, may turn into a threatening condition for both the mother and her baby. Most likely the development of gestational diabetes reflects individual predisposition [2] because, in spite of common restoration of normal glucose tolerance upon delivery, a large percentage of these women will develop overt type 2 diabetes later in life [3].

The importance of effective and timely diagnosis has been recognized and diagnostic criteria have been established. According to the criteria proposed by Carpenter and Coustan, gestational diabetes is diagnosed whenever, in response to a 3-hour OGTT, two abnormal glucose levels are recorded [4]. These criteria have been recently revisioned, following the report of the HAPO study [5] because there was concern that full assessment of risk was not be sufficiently disclosed by old criteria. For instance, we have recently shown that there is no much difference in insulin sensitivity or insulin secretion between GDM women and those who have only one abnormal value (i.e., not diagnostic for GDM) [6]. These alterations were particularly apparent for elevations of the 1-hour plasma glucose than it was for other OGTT time points, suggesting that this value may provide a better parameter for risk stratification. In order to assess this hypothesis, we have reanalysed our cohort of pregnant women by assessing changes in indexes of insulin action and secretion as a function of changes of 1-hour plasma glucose concentration in response to a 100 g OGTT.

## 2. Research Design and Methods

The study was performed on the same cohort of our previous observation [6]. Briefly, a total of 4,053 pregnant women with positive glucose challenge test (GCT: plasma glucose value ≥140 mg/dL 1 hr after a standard 50 g glucose load) carried out around the 27th week of gestation underwent a 3 h 100 g OGTT (samples: 0′, 1 hr, 2 hr, 3 hr) for determination of plasma glucose and insulin concentrations.

On the morning of the test, demographic, anthropometric, and clinical data were recorded. Glucose tolerance was defined according to the criteria of Carpenter and Coustan [4]. GDM was diagnosed when two or more plasma glucose levels exceeded the cutoff values; women with a single altered value were classified as having OAV and women not meeting any cutoff values were considered normotolerant.

In the current analysis we have arbitrarily divided the study population based on 20 mg/dL increment of plasma glucose concentration at 1-hour OGTT generating 5 groups (<120 mg/dL, *n* = 661; 120–139 mg/dL, *n* = 710; 140–159 mg/dL, *n* = 912; 160–179 mg/dL, *n* = 885; and ≥180 mg/dL *n* = 996).

The study was approved by local ethics committees and women gave their written informed consent to the collection of information, from their medical records.

### 2.1. Measurements and Statistical Analysis

Plasma glucose levels were determined on a Beckman Glucose Analyzer 2 (Beckman, Fullerton, CA) based on the glucose oxidase method and plasma insulin concentrations were measured by radioimmunoassay (INSI-CTK Irma; Dia Sorin). The inter- and intraassay coefficients of variation for all parameters were ≤5%.

Incremental areas under the glucose curve (AUC_gluc_) and insulin curve (AUC_ins_) during the OGTT were calculated using the trapezoidal rule. As a measure of insulin secretion, basal insulin and glucose concentrations were used for the estimation of *β*-cell secretion according to the homeostasis model assessment (HOMA-B) [7]: (20 × Ins_0_)/(Gluc_0_  −  3.5). HOMA-R index was calculated [7] to reflect insulin action in a manner independent of OGTT responses. We computed AUC_ins_/AUC_gluc_ as generalized insulinogenic index.

Data are given as percentages or mean ± SD. ANOVA with post hoc Bonferroni analysis was used to assess univariate differences among continuous variables; for qualitative variables, we used the *χ* ^2^ test to compare observed frequency between groups. All statistical comparisons were considered significant at *P* < 0.05. Statistical analyses were performed using a statistical package (Statview SE; SAS Institute, Cary, NC) on a Macintosh computer (Apple, Cupertino, CA).

## 3. Results

**Figure 1:**
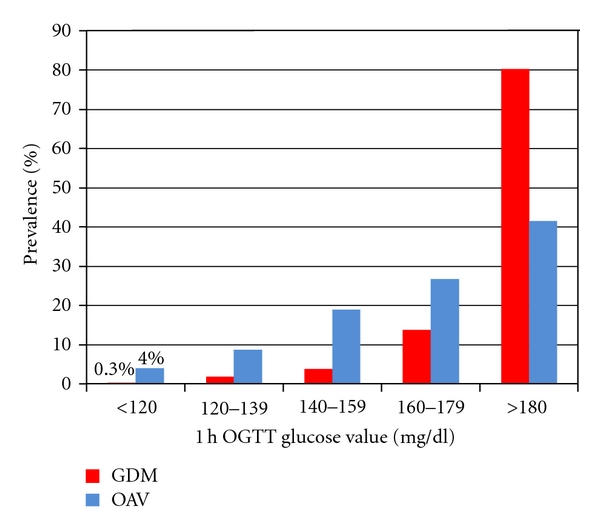
GDM and OAV prevalence according to 1-hour OGTT plasma glycaemia. GDM: gestational diabetes mellitus; OAV: women with one abnormal value during OGTT. The prevalence of GDM and OAV increases in the five groups of pregnant women (both *P* < 0.0001).

**Figure 2:**
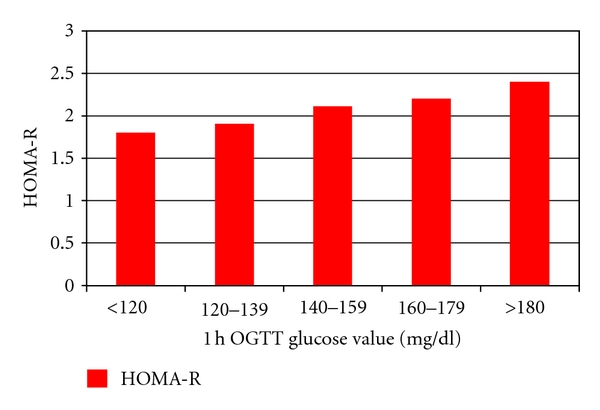
HOMA-R index according to 1-hour OGTT plasma glycaemia. HOMA-R index is estimated using the formulas proposed by Matthews et al. [7].

**Figure 3:**
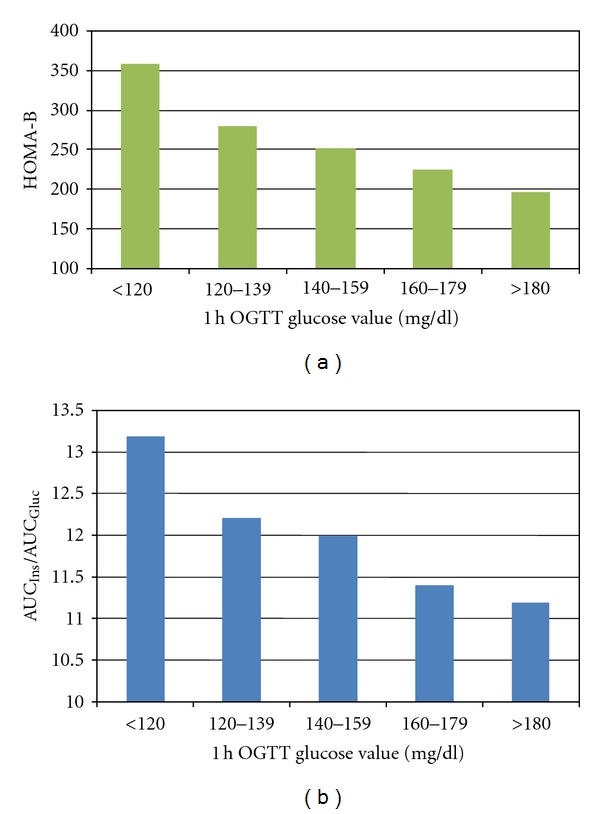
HOMA-B index and AUC_ins_/AUC_gluc_ according to 1-hour OGTT plasma glycaemia. HOMA-B index is estimated using the formulas proposed by Matthews et al. [7].

The main clinical characteristics of the 5 groups of ascending 1-hour plasma glucose are shown in Table 1. These 5 groups were comparable for all parameters with the exception of BMI although absolute differences did not exceed 1.2 kg/m^2^. Of interest, no difference was apparent in body weight gain during pregnancy among the 5 groups. The prevalence of GDM (0.3, 1.8, 3.9, 13.7, 80.3%) and that of one abnormal value (including 1-hour, 2-hour, 3-hour OAV) progressively increased in the 5 groups of pregnant women (both *P* < 0.0001, Figure 1). Fasting glucose and insulin levels increased among the 5 groups as shown in Table 2 (*P* < 0,0001; *P* < 0,01, resp.). Accordingly, both AUC_gluc_ and AUC_ins_ progressively increased over the spectrum of 1-hour plasma glucose concentrations (AUC_gluc_ from 8894 ± 1295 mg/dL/min to 14493 ± 1841 mg/dL/min, AUC_ins_ from 6562 ± 3274 pmol/l/min to 9150 ± 5516 pmol/l/min; both *P* < 0.0001 for trend). Moreover, HOMA-R increased in a linear manner from the group with the lowest to the one with the highest 1-hour plasma glucose level (*P* < 0,001) (Figure 2). HOMA-B progressively declined (*P* < 0.001) over the entire spectrum (Figure 3(a)). AUC_ins_/AUC_gluc_ decreased in a linear manner across the 5 groups (from 13, 19 ± 6.59 to 11,24 ± 6.53, *P* < 0,001, Figure 3(b)). When the analysis was restricted to groups with 1-hour plasma glucose <180 mg/dL, the progressive nature of defects in insulin secretion (HOMA-B: −29.7%) and insulin sensitivity (HOMA-R: +15%) were still apparent (all *P* < 0.001). 

## 4. Discussion

Although gestational diabetes is a well-recognized condition that may affect maternal and foetus health state, evidence is mounting that nondiabetic abnormalities of glucose homeostasis should be looked at with some caution [8, 9]. Carr et al. [10] have shown that pregnant women with OAV have twofold greater risk for subsequent diabetes as compared to women with no abnormal values in response to an OGTT. More recently, the HAPO study (Hyperglycemia and Adverse Pregnancy Outcome) [5], a clinical trial conducted on a large cohort of pregnant women aimed at clarifying the risk of adverse outcomes associated with various nondiabetic degrees of maternal glucose intolerance, showed a strong, continuous association between maternal glucose levels and increased birth weight as well as poor pregnancy outcomes. Moreover, among pregnant women with OAV, the alteration of 1-hour plasma glucose seems to provide the best predictive effect in terms of both pathophysiologic involvement and clinical outcome as initially highlighted by Retnakaran and colleagues [11]. In their paper they showed that all measures of severity of glycemic control were highest in women with GDM group, followed by the 1-hour OAV, 2-hour or 3-hour OAV, and NGT groups. Consistent with this finding, insulin sensitivity was highest in the NGT group and worsened in a progressive manner through the 2-hour or 3-hour OAV, 1-h OAV, and GDM groups. In our previous study, we showed that pregnant women with 1-hour OAV also had a greater impairment in their measures of *β*-cell function [6]. Moreover, pregnant women with 1-hour OAV, compared with those with 2-hour or 3-hour OAV, have a higher prevalence of adverse obstetric outcome, including caesarean delivery, macrosomia, and hypertensive disorder [12]. Finally, 3 months after delivery, women with 1-hour OAV tend to have persistent metabolic dysfunction, including higher plasma glucose levels, greater insulin resistance, and poorer *β*-cell function, very much alike women with prior GDM [13].

In summary, 1-hour OAV in pregnant women may be used for the stratification of the pregnancy risk. In order to gain further insights in the significance of 1-hour glucose, we have reanalyzed data from a large cohort of pregnant women showing that the progressive increase in this parameter is associated with a progressive loss of *β*-cell function as well as a decline in insulin sensitivity. Moreover the deterioration of the two main homeostatic parameters becomes apparent well within the so-called normal range of glucose tolerance in pregnancy. We would underline that in the same range (i.e., 1 h glucose values <120 mg/dL) GDM (0,3%) and OAV (4%) were also present and their prevalence progressively increases according to 1 h glucose values. These data suggest that even in categories with normal or very low 1-hour glucose values an alteration in glucose homeostasis is already apparent.

This also raises the question on the reason for 1-hour plasma glucose value to be so linked to degeneration of glucose homeostatic mechanisms. Although not directly assessed in the present study, it has already been reported that in nondiabetic individuals, the progressive increase of plasma glucose concentration at 30–60 min after an OGTT is dependent on *β*-cell function and insulin sensitivity in peripheral tissues [14, 15]. Therefore, it is plausible that development of insulin resistance in the liver and in the skeletal muscle and concomitant weakening of insulin secretion will result in progressive elevation of 1-hour glucose concentration, as suggested by our results. Moreover, in nondiabetic individuals it has been shown that 1-hour plasma glucose concentration has a stronger correlation with surrogates measures of hepatic and muscle insulin resistance and *β*-cell dysfunction compared with 2-hour plasma glucose levels [16].

The 1-hour plasma glucose value has been advocated as a risk marker not only in pregnant women. Abdul-Ghani et al. [17] have shown that the plasma glucose concentration at 1-hour after an OGTT is a powerful predictor of future risk for type 2 diabetes. He was also proposing a cutoff point of 155/mL dL to stratify diabetes risk. Moreover, Succurro et al. [18] have recently shown that individuals with normal glucose tolerance but a 1-hour plasma glucose levels ≥155 mg/dL have an atherogenic profile similar to IGT subjects. Obviously, we cannot extrapolate the continuous relationship between 1-hour plasma glucose levels and alterations in insulin action and secretion to the general population, but the strong similarities existing between GDM and IGT/type 2 diabetes are worth being considered. Our data, indeed, strongly suggest that alterations in glucose homeostasis related to increasing *t* 1-hour plasma glucose value are continuous variables: for each 20 mg/dL increase in 1-hour plasma glucose concentration there is a concomitant impairment in insulin action and a reduction in insulin secretion.

A limitation of our study should be represented by the indexes we used to determine insulin secretion and action that involve only basal glucose and insulin concentration; anyway, these indexes decrease as 1 h glucose value increases.

In conclusion, this study underlines the concept that the spectrum of glucose tolerance in pregnancy identifies a continuum of disturbance of glucose homeostasis and that 1-hour glucose level after 100 g OGTT may be considered a relevant marker for improving risk stratification in pregnant women. Moreover, although new IADPSG criteria for GDM diagnosis [19] erase the difference between GDM and OAV, the importance of 1-hour glucose level impairment still remains and our data reinforce the concept that the new lower diagnostic criteria will be useful in reducing the risk for short- (obstetrical) and long-term (metabolic and cardiovascular) complications.

## Figures and Tables

**Table 1:** Clinical features of pregnant women related to 1-hour glucose values during OGTT (data are means ± SD).

1 h glycaemia (mg/dL)	<120	120–139	140–159	160–179	≥180	ANOVA (*P*)
*N*	661	710	912	885	886	
Age (years)	30.7 ± 4	31.1 ± 4	31.7 ± 4	32.1 ± 4	32.2 ± 4	NS
Prepregnancy weight (kg)	64.9 ± 10	63.5 ± 9	64.3 ± 11	65.1 ± 12	66.2 ± 12	<0.001
Prepregnancy BMI (kg/m^2^)	24.4 ± 3.8	23.7 ± 3.6	24 ± 4.2	24.3 ± 4.6	24.9 ± 4.4	<0.01
Weight gain (kg)	7.5 ± 3.4	7.7 ± 3.3	7.5 ± 3.3	7.7 ± 3.5	7.7 ± 3.6	NS
Systolic BP (mmHg)	116.8 ± 11	115.1 ± 10	116 ± 12	114.8 ± 12	116.7 ± 12	NS
Diastolic BP (mmHg)	71.9 ± 8	71.4 ± 8	71.2 8	71.1 ± 8	71.7 ± 8	NS
Total cholesterol (mg/dL)	261 ± 45	258 ± 46	257 ± 44	254 ± 39	263 ± 50	NS
LDL cholesterol (mg/dL)	160 ± 43	179 ± 32	162 ± 36	166 ± 45	166 ± 40	NS
HDL cholesterol (mg/dL)	54.3 ± 18	58.3 ± 18	59.2 ± 20	61.1 ± 18	62.2 ± 19	NS
Triglycerides (mg/dL)	199 ± 67	205 ± 81	201 ± 66	191 ± 79	197 ± 77	NS

**Table 2:** Fasting glucose and insulin levels related to 1-hour glucose values during OGTT (data are means ± SD).

1 h glycaemia (mg/dL)	<120	120–139	140–159	160–179	≥180	ANOVA (*P*)
Fasting glucose (mg/dL)	72.4 ± 10.4	80.8 ± 9.14	82.11 ± 9.16	84.1 ± 10	87.5 ± 11	<0.0001
Fasting insulin (pmol/L)	55.19 ± 29.5	57.5 ± 36.6	61.8 ± 47	63.5 ± 53	67.7 ± 51	<0.01

## Abbreviations

OGTT: Oral glucose tolerance test
1 h OGTT: 1-hour during OGTT
GDM: Gestational diabetes
OAV: One abnormal value
AUC_gluc_: Area under glucose curve
AUC_ins_: Area under insulin curve
HOMA-B: Index of insulin secretion (homeostasis model assessment: *β* cell function)
HOMA R: Index of insulin sensitivity (homeostasis model assessment:insulin resistance).
